# Virological effectiveness and CD4+ T-cell increase over early and late courses in HIV infected patients on antiretroviral therapy: focus on HCV and anchor class received

**DOI:** 10.1186/1742-6405-9-18

**Published:** 2012-06-15

**Authors:** Davide Motta, Nigritella Brianese, Emanuele Focà, Paola Nasta, Franco Maggiolo, Massimiliano Fabbiani, Giuliana Cologni, Simona Di Giambenedetto, Massimo Di Pietro, Nicoletta Ladisa, Laura Sighinolfi, Silvia Costarelli, Filippo Castelnuovo, Carlo Torti

**Affiliations:** 1 Institute of Infectious and Tropical Diseases, University of Brescia, Brescia, Italy; 2 Department of Infectious Diseases, Ospedali Riuniti, Bergamo, Italy; 3 Institute of Clinical Infectious Diseases, Catholic University of Sacred Heart, Rome, Italy; 4 Infectious Diseases Clinic, Santa Maria Annunziata Hospital, Florence, Italy; 5 Clinic of Infectious Diseases, Policlinico di Bari, Bari, Italy; 6 Department of Infectious Diseases, S. Anna Hospital, Ferrara, Italy; 7 Department of Infectious Diseases, Istituti Ospitalieri, Cremona, Italy; 8 Spedali Civili di Brescia, Brescia, Italy; 9 Unit of Infectious Diseases, University “Magna Graecia”, Catanzaro, Italy

**Keywords:** HIV, HCV, HAART

## Abstract

**Background:**

The aim of this study was to explore the effects of HCV co-infection on virological effectiveness and on CD4+ T-cell recovery in patients with an early and sustained virological response after HAART.

**Methods:**

We performed a longitudinal analysis of 3,262 patients from the MASTER cohort, who started HAART from 2000 to 2008. Patients were stratified into 6 groups by HCV status and type of anchor class. The early virological outcome was the achievement of HIV RNA <500 copies/ml 4–8 months after HAART initiation. Time to virological response was also evaluated by Kaplan-Meier analysis. The main outcome measure of early immunological response was the achievement of CD4+ T-cell increase by ≥100/mm^3^ from baseline to month 4–8 in virological responder patients. Late immunological outcome was absolute variation of CD4+ T-cell count with respect to baseline up to month 24. Multivariable analysis (ANCOVA) investigated predictors for this outcome.

**Results:**

The early virological response was higher in HCV Ab-negative than HCV Ab-positive patients prescribed PI/r (92.2% *versus* 88%; *p* = 0.01) or NNRTI (88.5% *versus* 84.7%; *p* = 0.06). HCV Ab-positive serostatus was a significant predictor of a delayed virological suppression independently from other variables, including types of anchor class. Reactivity for HCV antibodies was associated with a lower probability of obtaining ≥100/mm^3^ CD4+ increase within 8 months from HAART initiation in patients treated with PI/r (62.2% among HCV Ab-positive patients *versus* 70.9% among HCV Ab-negative patients; *p* = 0.003) and NNRTI (63.7% *versus* 74.7%; *p* < 0.001). Regarding late CD4+ increase, positive HCV Ab appeared to impair immune reconstitution in terms of absolute CD4+ T-cell count increase both in patients treated with PI/r (*p* = 0.013) and in those treated with NNRTI (*p* = 0.002). This was confirmed at a multivariable analysis up to 12 months of follow-up.

**Conclusions:**

In this large cohort, HCV Ab reactivity was associated with an inferior virological outcome and an independent association between HCV Ab-positivity and smaller CD4+ increase was evident up to 12 months of follow-up. Although the difference in CD4+ T-cell count was modest, a stricter follow-up and optimization of HAART strategy appear to be important in HIV patients co-infected by HCV. Moreover, our data support anti-HCV treatment leading to HCV eradication as a means to facilitate the achievement of the viro-immunological goals of HAART.

## Introduction

The introduction of highly-active anti-retroviral therapy (HAART) has determined a dramatic improvement in the management of HIV-1 infection, leading to suppression of HIV viraemia and consequent restoration of the immune function. However, viro-immunological effectiveness of HAART may be hampered by other factors [1-4].

In particular, the impact of co-infection with hepatitis C virus (HCV) on treatment response is still controversial despite the large number of published data. In this respect, co-infection with HCV has a potential great relevance because its prevalence is up to 80% in those who acquired HIV through injection drug use [5].

Several studies reported a reduced CD4+ T-cell count recovery in patients with HIV and HCV co-infection with respect to patients with HIV alone [6-13]. This conclusion was further supported by a meta-analysis [14], but was not confirmed by other results [15-17]. Moreover, variable levels of HIV RNA may confound the association of HIV co-infection with CD4+ T-cell count increase. In particular, we need data in patients who obtained early and sustained virological response to minimize the influence of variable levels of HIV replication on the immunological response because the results currently available are inconsistent, with some studies demonstrating a difference [10] and others disproving it [18,19].

Regarding virological response, several studies did not demonstrate any significant associations with HCV status [16,17], however one recent study evaluated time to virological suppression as primary outcome and found that HCV Ab-positivity was associated with a reduced rate of virological suppression at bivariate analysis, but not adjusting for history of intravenous drug use. [20].

The aim of this study was to explore the effects of HCV co-infection on virological effectiveness and on CD4+ T-cell reconstitution in patients with an early and sustained virological response after HAART. Also, stratified analyses were conducted by anchor drug --i.e. the antiretroviral drug added as third component to a backbone of nucleoside reverse transcriptase inhibitors-- to explore whether different drug characteristics may have an effect.

## Methods

### Design of the study and cohort used

We conducted an analysis on naïve patients enrolled in the Italian MASTER (Management of standardized antiretroviral therapy) cohort who started first line HAART containing protease inhibitors (either boosted by ritonavir - PI/r - or not boosted) or non-nucleoside reverse transcriptase inhibitor (NNRTI) from 1^st^ January 2000 to 31^st^ December 2008. Patients were stratified into 6 groups according to the type of anchor class received (PI, PI/r or NNRTI) and HCV Ab status (positive or negative).

The MASTER cohort is a longitudinal multicenter cohort consisting of a general HIV patient population in nine referral centres throughout Italy (http://www.mastercohort.it). The distinguishing characteristic of this cohort is that data are compiled in a common electronic chart (Health & Notes 3.5®, Healthware S.p.A., Naples, Italy) in use in the participating centres. Data are recorded over a standardized time-scale every 3 months, with merging and data cleaning performed at the coordinating centre every 6 months. Subjects gave written informed consent for participation in the observational cohort, and each site obtained approval by its Ethics Committee.

### Study outcomes

#### Virological outcome

The main virological outcome was the achievement of early virological response, i.e., HIV RNA <500 copies/ml within 8 months from HAART initiation (values included between 4 and 8 months, closest to month 8, were considered). This limit of detection for undetectability was chosen because it was the highest value ever used in the clinical centers participating to the MASTER cohort during the time frame of the study. The main virological outcome was assessed both through intent-to-treat (ITT) and on treatment (OT) analyses. At ITT analysis, all patients were included even if they changed drugs used as anchor (PI or PI/r to NNRTI and *vice-versa*, or PI to PI/r and *vice-versa*); at OT analysis, only patients who did not change class therapy were considered. A logistic model was performed with undetectable HIV RNA as dependent variable and HIV RNA at baseline and HCV Ab reactivity as independent variables.

We also assessed the rate of patients with HIV RNA <500 copies/ml by Kaplan Meier survival curves over a mean length of follow-up of 52 (SD: 33) months.

Possible predictors of time to virological suppression were assessed through Cox regression analysis considering as independent variables either baseline factors (age at HAART initiation, gender, risk factors for HIV acquisition, history of AIDS diagnosis, HIV RNA, HCV Ab status, calendar year at HAART) or time dependent factors (CD4+ T-cell count, type of anchor class). All the variables were input in a multivariate model. The proportional hazards assumption was checked by determining whether plotting the log [−log(survival)] curve against survival time resulted in parallel lines for each covariate. Moreover, scaled Schoenfeld residuals were plotted on functions of time and tested for a non-zero slope.

#### Immunological outcomes

For the study of early immunological response, we used patients who obtained HIV RNA <500 copies/ml within 8 months from HAART initiation (values included between 4 and 8 months, closest to month 8, were considered) and CD4+ T-cell count was assessed in the same time frame (values closest to month 8 were used). In these patients, two immunological outcomes were evaluated: (i) CD4+ T-cell increase by ≥100/mm^3^ from baseline (categorical outcome); (ii) absolute CD4+ T-cell change (continuous outcome). For the categorical outcome, a bivariate logistic analysis was performed with HIV RNA at baseline and HCV Ab status (positive or negative) used as independent variables. For the continuous outcome, ANCOVA model was performed. In this analysis, CD4+ T-cell change from baseline was the dependent variable, HIV RNA value at baseline was the covariate, and HCV Ab reactivity was the fixed effect.

For the study of late immunological response, the same patients were studied until HIV RNA remained undetectable (<500 copies/ml), patients were lost to follow-up (lack of exams for ≥6 months), or died. Moreover, analyses were truncated when <30% patients were under observation to avoid inconsistent results due to an insufficient number of patients under study.

We studied the immunological response as absolute CD4+ T-cell count increase adjusted for CD4+ T-cell count at baseline. First, ANCOVA model was performed in order to assess differences between HCV Ab-positive and HCV Ab-negative patients, stratified by anchor class at month 24 after HAART initiation. In this analysis, baseline CD4+ T-cell count and HCV Ab reactivity were fixed effects. Second, ANCOVA models were used with absolute CD4+ T-cell count increase as dependent variables to assess predictors at months 12 and 24. At this analysis, we considered as possible predictors: age at HAART initiation, gender, risk factors for HIV acquisition, history of AIDS diagnosis, HIV RNA, CD4+ T-cell count, HCV Ab status, calendar year at HAART and type of anchor class.

### Additional statistical notes

Descriptive statistics were calculated for quantitative variables (mean, standard deviation – SD, median, minimum and maximum) and qualitative variables (absolute and percent frequencies) as appropriate. Ninety-five percent confidence intervals (95%CI) were calculated for appropriate cases. Variables were compared by means of F test from ANOVA model or *χ*^2^ test in case of quantitative or qualitative variables, respectively.

All analyses have been performed using the statistical software package SAS 9.2 (SAS Inc. Cary, NC). All *p* value were two-tailed and considered significant if <0.05.

## Results

We studied a cohort of 3,262 patients, of whom 863 were positive for HCV Ab. Main characteristics of the population at baseline, stratified into 6 groups by HCV Ab status and type of anchor class (PI, PI/r, NNRTI), are shown in Table 1. In the whole population males were 73%, mean age was 37.2 years at cohort entry and was 39.1 years at HAART initiation. HCV Ab-positive patients were more likely to be younger and IVDU than HCV Ab-negative patients; AIDS events at baseline were more frequent in HCV Ab-negative patients.

**Table 1:** Patients’ characteristics at baseline

***Variable***	***HCV Ab+*** ***PI*** ***(n = 146)***	***HCV Ab-*** ***PI*** ***(n = 208)***	***HCV*** ***Ab + PI + r*** ***(n = 365)***	***HCV Ab-*** ***PI + r*** ***(n = 1059)***	***HCV*** ***Ab + NNRTI*** ***(n = 352)***	***HCV Ab-*** ***NNRTI*** ***(n = 1132)***	***p***
Males [N(%)]	112 (76.7)	148 (71.2)	284 (77.8)	751 (70.9)	257 (73.0)	829 (73.2)	0.163
Age at cohort entry, years [Mean (SD)]	34.3 (8.1)	38.3 (10)	37.2 (9.3)	38.4 (10.6)	34.3 (8.7)	37.2 (10.1)	<0.001
Age at HAART initiation, years [Mean (SD)]	37.8 (6.2)	39.0 (9.6)	41.2 (7.6)	39.5 (10.4)	38.3 (7.3)	38.5 (9.9)	<0.001
Calendar year at HAART [Median (range)]	2001 (2000–2009)	2001 (2000–2009)	2006 (2000–2009)	2006 (2000–2009)	2003 (2000–2009)	2004 (2000–2010)	
Risk factors for HIV acquisition [N(%)]							
Heterosexual	24 (16.4)	122 (58.7)	74 (20.3)	587 (55.4)	79 (22.4)	678 (59.9)	<0.001
MSM	6 (4.1)	56 (26.9)	18 (4.9)	254 (24)	26 (7.4)	316 (27.9)	
IVDU	110 (75.3)	10 (4.8)	228 (62.5)	21 (2)	234 (66.5)	27 (2.4)	
Other/unknown	6 (4.1)	20 (9.6)	45 (12.3)	197 (18.6)	13 (3.7)	111 (9.8)	
AST, IU/L [Mean (SD)]	69.3 (56.4)	44.9 (96.9)	62.1 (54.7)	32.5 (24.2)	58.0 (47.9)	34.0 (28.0)	<0.001
ALT, IU/L [Mean (SD)]	68.9 (61.7)	48.2 (88.5)	70.6 (70.1)	40.9 (45.4)	64.5 (51.4)	38.9 (39.3)	<0.001
Serum creatinine, mg/dL[Mean (SD)]	0.79 (0.26)	1.00 (1.35)	0.84 (0.45)	0.85 (0.43)	0.82 (0.26)	0.87 (0.64)	0.017
Cholesterolaemia, mg/dL [Mean (SD)]	138.3 (40.3)	172.0 (49.7)	150.1 (42.6)	167.1 (46.4)	156.7 (40.3)	165.1 (39.5)	<0.001
Nadir of CD4+ T-cells, cells/mm^3^ [Mean (SD)]	143.1 (122.5)	144.8 (140.6)	169.5 (123.6)	169.5 (133.3)	179.3 (115.9)	198.5 (118.8)	<0.001
AIDS diagnosis [N (%)]	43 (29.5)	76 (36.5)	95 (26.0)	314 (29.7)	67 (19.0)	215 (19.0)	<0.001
CD4+ T-cell count, cells/mm^3^ [Mean (SD)]	222.4 (220.1)	188.3 (198.4)	210.1 (157.3)	202.5 (175.8)	239.6 (146.5)	233.8 (144.1)	<0.001
HIV-1 RNA, copies/ml [Mean (SD)]	143,968 (178,492)	187,909 (192,419)	208,367 (397,492)	225,939 (529,767)	156,582 (260,891)	191,210 (543,678)	0.116

N: number, SD: standard deviation, PI/r: protease inhibitors boosted by ritonavir, PI: protease inhibitors not boosted by ritonavir NNRTI: non-nucleoside reverse transcriptase inhibitor, IVDU: intravenous drug users, HAART: highly active antiretroviral therapy, MSM: men who have sex with men, AST: Aspartate aminotransferase, ALT: Alanine aminotransferase.

### Virological analysis (patients stratified by HCV serostatus and classes of anchor drugs)

The early virological response rate was significantly higher in HCV Ab-negative than in HCV Ab-positive patients. The ITT analysis showed that, in the overall population, 87.8% patients achieved virological success. In patients receiving a PI (N = 354), those who did not reach an early virological response were 34/146 (23.3%) among HCV Ab-positive patients *versus* 52/208 (25%) HCV Ab-negative patients (*p* = 0.66); among the 1424 patients treated with PI/r, 44/365 (12%) HCV Ab-positive *versus* 83/1059 (7.8%) HCV Ab-negative patients did not reach the virological response (OR 0.612, 95%CI 0.416 to 0.903, *p* = 0.01); in the NNRTI group (N = 1484) those who did not achieve the virological response were 54/352 (15.3%) HCV Ab-positive *versus* 130/1132 (11.5%) HCV Ab-negative patients (OR 0.717, 95%CI 0.509 to 1.01, *p* = 0.06). Also at OT analysis, significant differences between HCV Ab-positive and HCV Ab-negative patients emerged only in those treated with PI/r (OR 0.582, 95%CI 0.389 to 0.869, *p* = 0.01), and in those treated with NNRTI (OR 0.618, 95%CI 0.419 to 0.912, *p* = 0.02).

**Figure 1:**
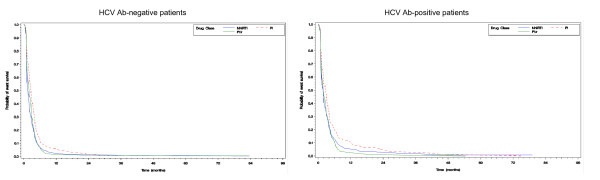
**Kaplan Meir analysis of time to virological response.** Time to HIV-1 RNA <500 copies/ml in HCV Ab-negative patients (panel a) and HCV Ab-positive patients (panel b) by classes of anchor drugs.

Figure 1 (panels “a” and “b”) shows the Kaplan-Meier analyses in HCV Ab-negative and in HCV Ab-positive patients for the achievement of virological suppression. Only in patients treated with NNRTI, there was a statistically significant difference for the time to achieve virological response being shorter in HCV Ab-negative patients (log-rank *p* = 0.003). By contrast, no statistically significant differences were found in patients treated with PI and, among patients receiving PI/r, a borderline statistical significance was found in favor of the HCV Ab-negative group (log-rank *p* = 0.096).

### Predictors of time to achieve virological suppression

**Figure 2:**
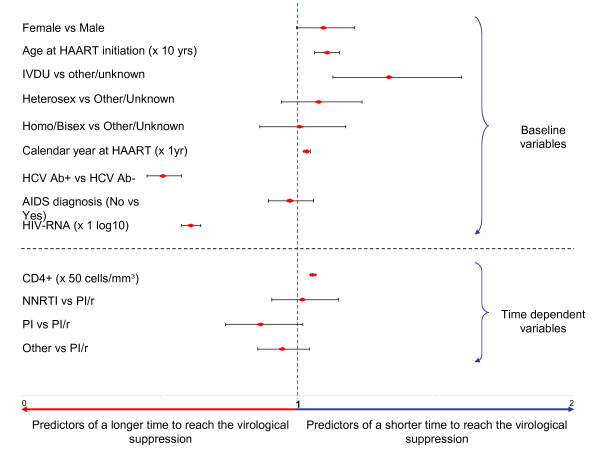
**Predictors of time to undetectable HIV RNA (< 500 copies/ml).** HAART: highly active antiretroviral therapy, IVDU: intravenous drug users, NNRTI: non-nucleoside reverse transcriptase inhibitor, PI/r: protease inhibitors boosted by ritonavir, PI: protease inhibitors not boosted by ritonavir.

Cox regression analysis was performed in order to explore possible predictors of time to achieve virological suppression (Figure 2). We found that, among the baseline variables, age at HAART initiation (x 10-years older) (HR 1.109, 95%CI 1.065 to 1.155, *p* < 0.001), IVDU as risk factors for HIV acquisition (HR 1.344, 95%CI 1.130 to 1.599, *p* = 0.001), calendar year at HAART (x 1-year more recent) (HR 1.035, 95%CI 1.023 to 1.046, *p* < 0.001), were predictors of shorter time to reach the virological success; instead, HCV Ab-positive status (HR 0.738, 95%CI 0.652 to 0.834, *p* < 0.001) and higher level of HIV RNA (x1 log_10_ copies/ml) (HR 0.882, 95%CI 0.832 to 0.935, *p* < 0.001) predicted a longer time to obtained the same outcome. Among time-dependent variables, only increasing CD4+ T-cell count was directly associated with time to achieve the virological outcome (HR 1.057, 95%CI 1.047 to 1.066, *p* < 0.001), while type of HAART was not (NNRTI *versus* PI/r, *p* = 0.75; PI *versus* PI/r, *p* = 0.08; others *versus* PI/r, *p* = 0.27).

### Immunological analysis (patients stratified by HCV serostatus and classes of anchor drugs)

For the early immunological response, achievement of CD4+ T-cell increase ≥100/mm^3^ within 8 months from HAART initiation was evaluated among patients with a virological response. In this analysis, 2,682 patients who had values of CD4+ T-cell count both at baseline and after 4–8 months were considered. Among patients receiving a PI (N = 239) there were not significant differences between mono- and co-infected patients at bivariate analysis (60.8% *versus* 65.5%, *p* = 0.35). Conversely, in the PI/r group (N = 1,220) HCV Ab-positive patients had a lower probability to reach the outcome than HCV Ab-negative patients (62.2% *versus* 70.9%, OR 0.662, 95%CI 0.502 to 0.872, *p* = 0.003). Also, among NNRTI-receiving patients (N = 1,223), CD4+ T-cell count increase ≥100/mm^3^ was less likely to be achieved by HCV Ab-positive patients (63.7% *versus* 74.7%, OR 0.595, 95%CI 0.445 to 0.797, *p* < 0.001). A significant difference in CD4+ T-cell count increase was also found in this group at ANCOVA when absolute CD4+ T-cell count increase was analyzed as continuous measure (−36.8/mm^3^, 95%CI −53.6 to −20.1, *p* < 0.001).

**Figure 3:**
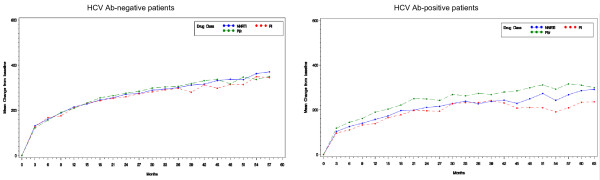
**Immune-recovery evaluated as absolute CD4+ T-cell count increase with respect to the baseline.** Late immunological outcomes in patients who obtained sustained HIV RNA suppression by year 1: absolute CD4+ T-cell count increase in HCV Ab-negative patients (panel a) and in HCV Ab-positive patients (panel b). Note that for immunological analyses data were cut at month 63 when 30% of the enrolled patients were still under observation.

For the late immunological response, the absolute CD4+ T-cell count for both HCV Ab-negative and HCV Ab-positive patients is depicted in Figure 3. By ANCOVA, at month 24 there was a significant difference between HCV Ab-positive and HCV Ab-negative patients with sustained virological success in terms of absolute CD4+ T-cell count increase both in patients treated with PI/r (mean change [SD]: 248.4 cells/mm^3^ [188.6] in HCV Ab-positive patients *versus* 275.5 cells/mm^3^ [203.4] in HCV Ab-negative patients; *p* = 0.013) and in those treated with NNRTI containing regimens (mean change [SD]: 211.1 cells/mm^3^ [168.3] in HCV Ab-positive patients *versus* 271.0 cells/mm^3^ [183.4] in HCV Ab-negative patients; *p* = 0.002).

### Predictors of CD4+ T-cell count recovery

Table 2 shows the results of the ANCOVA model performed to investigate possible predictors of CD4+ T-cell recovery at different time-point of the study.

**Table 2:** Predictors of immune recovery (absolute CD4+ T-cell increase from baseline after HAART)

	**12 months**		**24 months**	
***Variable***	**Estimate (SE)**	***p***	**Estimate (SE)**	***p***
Age at HAART initiation [x10-yrs]	−13.6 (3.7)	0.002	−12.0 (5.1)	0.02
AIDS [no vs. yes]	N.S.		25.0 (11.3)	0.03
CD4+ [x50 cells/mm^3^]	N.S.		−11.5 (1.6)	<0.001
HIV RNA [x1 log_10_]	−46.8 (5.5)	<0.001	47.1(7.5)	<0.001
HCV + vs HCV-	−38.4 (11.0)	0.005	N.S.	
PI vs PI/r	N.S.		−33.4 (14.8)	<0.02

Results of the ANCOVA models with absolute CD4+ T-cell increase as dependent variable after 12 and 24 months from HAART initiation. Only variables that resulted to be statistically significant in at least one time-point are reported. Models were adjusted for age at HAART onset, gender, risk factors for HIV acquisition, history of AIDS, basal HIV RNA, CD4+ T-cell count, HCV Ab status, calendar year at HAART and type of treatment as anchor (PI/r vs NNRTI or PI).

HCV Ab-positive status appeared to predict smaller CD4+ T-cell increase after 12 months of HAART (*p* = 0.005), independently from age at HAART onset, and HIV RNA at baseline, both significant predictors of this outcome (*p* = 0.002 and <0.001, respectively). However, after 24 month of HAART, the impact of HCV Ab status disappeared.

## Discussion

In this study, the possible impact of HCV co-infection on viro-immunological effectiveness of different HAART regimens was explored both in the early and in the late follow-up and using both a stratified and a multivariable analysis.

In the overall population we found that 88% patients reached HIV RNA suppression within months 4–8 months from initiation of HAART at ITT analysis. However, percentages differed by HCV co-infection and by anchor class received. In fact, HCV Ab-positive patients obtained HIV RNA suppression less likely than HIV mono-infected patients, though this difference was greater in those treated with PI/r (88% *versus* 92.2%) and NNRTI (84.7% *versus* 88.5%) than in those treated with PI (76.7% *versus* 75%). It is possible that this finding reflects a treatment adherence phenomenon. In fact, on one hand, if one assumes that HCV Ab-positivity is a proxy for a negative behavior related to IVDU, with a negative influence on treatment adherence [5,21], the difference in virological suppression would be magnified in patients who received more complex regimens (such as the PI/r most frequently used at the time of the study) [22]. On the other hand, in patients prescribed less potent regimens (such as PI), differences between the two groups could have been diluted. Indeed, the rate of treatment success in these patients was the lowest.

However, when predictors of HIV RNA response were investigated, HCV co-infection appeared to be a negative predictor independently from IVDU, suggesting that the effects of these two variables were not entirely overlapping. This may be due either to HCV co-infection “*per se*” (probably affecting the immune control of HIV replication) or to adherence as unmeasured variable because HCV co-infected patients may have been less adherent to treatment even though they were not IVDU. Further bio-psycho-social investigations are needed to understand the complex relationship between HCV co-infection, the role of adherence behaviors and IVDU as risk factor.

Regarding the other predictors of virological success, the positive impact of aging (possibly reflecting better adherence in older patients) is not new [23,24], as well as the positive impact of more recent calendar years, possibly reflecting a significant improvement in the drug regimens and in the overall standard of care [25,26].

We found a positive impact of higher CD4+ T-cell count as a protective variable for achieving HIV RNA suppression. It is possible that the immune system (either at baseline or reconstituted by HAART) has a role in better controlling HIV replication. If this is the case, our results point to earlier HAART initiation as a means to preserve immune system so that virological suppression is more frequent. But also the lack of virological suppression (or virological suppression in presence of suboptimal adherence[27]), may be a cause rather than a consequence of lower CD4+ count, supporting the importance of a continuing assessment of treatment adherence, especially in HIV/HCV co-infected patients who appear to be disadvantaged.

Regarding the immunological outcomes, reactivity for HCV Ab was associated with a lower probability of obtaining ≥100 CD4+ T-cell count increase within 8 months from HAART initiation and with a smaller CD4+ T-cell count recovery up to 12 months in a multivariable analysis. It has been hypothesized that CD4+ T-cell depletion might result from ongoing cell activation and apoptosis driven by HCV [28] and therefore HCV could lower the CD4+ T-cell recovery through a direct pathogenic effect on lymphocytes even after HAART [29]. Our results, together with this interpretation, suggest that it is important to treat HCV infection aimed at obtaining its eradication, not only with the main goal of treating liver disease but also as a means to improve the viro-immunological outcomes of HAART, especially early upon its inception.

Greub et al. studied a group of 1596 patients (33% of them were HCV Ab-positive) with sustained HIV RNA <400 copies/ml and found that HCV Ab-positive subjects had an impaired CD4+ T-cell recovery in the long term [10]. By contrast, data from Kaufmann’s studies did not show any significant difference in CD4+ T-cell recovery when comparing HCV Ab-positive and HCV Ab-negative patients [18]. Our results suggest that, even though HCV infection may have an impact in the short term (either because immune activation has not yet been down-regulated or because HIV RNA is less effectively controlled early upon HAART), a more prolonged undetectability of HIV RNA may eventually counteract the apparent disadvantage occurring in HCV co-infected patients.

The present study suffers from limitations: first, HCV RNA was not considered, therefore patients with HCV Ab-positive but negative HCV RNA may have been included in the analysis, even if patients treated with anti-HCV drugs were excluded with the aim to reduce this possible bias. Second, HCV genotypes were not considered. A small study found that, among 171 HIV/HCV co-infected patients, those infected by HCV genotype 3 had inferior CD4+ T-cell increase after 12 months of follow-up [30]. However data from Antonucci et al. [7] did not find any correlation between HCV genotypes and immune-reconstitution in a cohort of 284 antiretroviral naïve patients. Therefore, further studies are needed to investigate the possible role of HCV genotypes on immune reconstitution in larger cohorts of patients over a longer follow-up. Third, other possible confounders such as severity of liver disease were not measured. Fourth, a <500 copies/ml cut-off for undetectability was considered. It is possible that persistent HIV replication below this cut-off may have had an impact on immune-reconstitution but this effect was not captured in our analysis.

In conclusion, the analysis of virological response suggests that HCV Ab-positive patients need focused interventions to maximize the effectiveness of HAART, especially early upon HAART initiation. Moreover, an higher CD4+ T-cell count predicted better virological control suggesting that preservation of immune functions is important not only to prevent clinical complications but also as a means to better control HIV replication. Thus, earlier HAART initiation is supported, especially in HIV/HCV co-infected subjects whose viro-immunological response may be compromised relative to HIV mono-infected patients. Lastly, it is possible that HCV eradication through specific treatments is beneficial not only for the main goal of treating liver disease but also for the viro-immunological effectiveness of HAART.

## Competing interests

Co-authors (CT, NB, EF, PN, FM, GC, SDG, MF, MDP, NL, LS, SC, FC) received grants from several Pharmaceutical Companies for participating to advisory board and scientific conferences but the received supports did not influence the content of this paper. Remaining co-authors: none to declare.

## Authors’ contributions

*Study concept and design:* CT, DM, NB. *Acquisition of data:* CT, DM, NB, EF, PN, FM, GC, SDG, MF, MDP, NL, LS, SC, FC. *Analysis and interpretation of data:* CT, DM, NB, EF. *Drafting of the manuscript:* CT, DM, NB. *Critical revision of the manuscript for important intellectual content:* EF, PN, FM, GC, SDG, MF, MDP, NL, LS, SC, FC. All authors read and approved the final manuscript.
